# Supplementary figures and images for: Subclinical Infection of Macaques and Baboons with A Baboon Simarterivirus
Source: Viruses. 2018 Dec 10;10(12):701. doi: 10.3390/v10120701 (PMC6316555; doi:10.3390/v10120701)

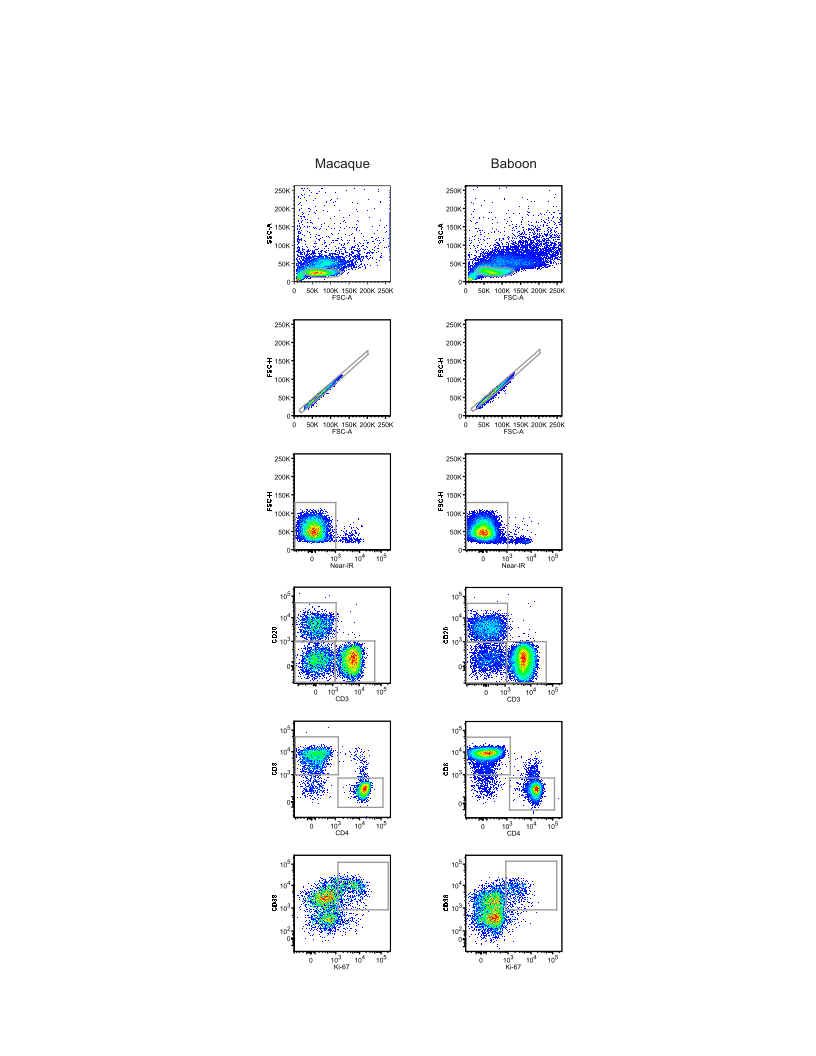

Supplement: Supplementary file 1 [file viruses-10-00701-s001.zip › supplementary/Fig S1. Flow macaque vs baboon.png]
